# The Embryonic Transcriptome of the Red-Eared Slider Turtle (*Trachemys scripta*)

**DOI:** 10.1371/journal.pone.0066357

**Published:** 2013-06-19

**Authors:** Nicholas J. Kaplinsky, Scott F. Gilbert, Judith Cebra-Thomas, Kersti Lilleväli, Merly Saare, Eric Y. Chang, Hannah E. Edelman, Melissa A. Frick, Yin Guan, Rebecca M. Hammond, Nicholas H. Hampilos, David S. B. Opoku, Karim Sariahmed, Eric A. Sherman, Ray Watson

**Affiliations:** 1 Department of Biology, Swarthmore College, Swarthmore, Pennsylvania, United States of America; 2 Department of Biology, Millersville University, Millersville, Pennsylvania, United States of America; 3 Department of Developmental biology, Institute of Molecular and Cell Biology, University of Tartu, Tartu, Estonia; 4 Department of Physiology, Faculty of Medicine, University of Tartu, Tartu, Estonia; Hospital for Sick Children, Canada

## Abstract

The bony shell of the turtle is an evolutionary novelty not found in any other group of animals, however, research into its formation has suggested that it has evolved through modification of conserved developmental mechanisms. Although these mechanisms have been extensively characterized in model organisms, the tools for characterizing them in non-model organisms such as turtles have been limited by a lack of genomic resources. We have used a next generation sequencing approach to generate and assemble a transcriptome from stage 14 and 17 *Trachemys scripta* embryos, stages during which important events in shell development are known to take place. The transcriptome consists of 231,876 sequences with an N_50_ of 1,166 bp. GO terms and EC codes were assigned to the 61,643 unique predicted proteins identified in the transcriptome sequences. All major GO categories and metabolic pathways are represented in the transcriptome. Transcriptome sequences were used to amplify several cDNA fragments designed for use as RNA *in situ* probes. One of these, *BMP5*, was hybridized to a *T. scripta* embryo and exhibits both conserved and novel expression patterns. The transcriptome sequences should be of broad use for understanding the evolution and development of the turtle shell and for annotating any future *T. scripta* genome sequences.

## Introduction

Over the past thirty years, the mechanisms that underlie the fundamental processes of animal development have been identified and characterized at a molecular level in a select group of model organisms. Although the field of embryology traditionally investigated a diverse range of organisms the full power of developmental genetics has been brought to bear on developmental questions in only a few animal model systems [1]–[3]. Elucidation of the mechanisms underlying the development of morphological structures which are not found in model systems has, until recently, been limited by a lack of genetic and genomic resources in non-model systems.

The turtle shell is an evolutionary novelty restricted to the order Chelonia that first appears in the fossil record 210MYA [4], [5]. The bony shell consists of the dorsal carapace and the ventral plastron. Each consists of a set of fused bones, some of which exist in other organisms and some of which are unique to turtles [6]. Understanding the evolution of the turtle shell involves answering fundamental questions about how new morphological structures develop. Did the evolution of the turtle shell require the innovation of new developmental programs or were existing programs modified in the Chelonians? If existing developmental programs were modified, which programs were recruited and how were they altered?

Work on shell formation in the red-eared slider turtle (*Trachemys scripta*) over the past decade suggests that the evolution of the turtle shell involved the co-option of highly conserved vertebrate developmental programs. The formation of the carapace represents a unique variation on vertebrate rib growth, coupled with existing programs of dermal ossification. The plastron originates in a different manner, as it appears to be derived from a late migrating population of neural crest cells, suggesting a similar origin for the plastron and facial bones [6].

The carapace is initiated by a bulge of mesodermal and ectodermal cells in the skin known as the carapacial ridge (CR). This turtle-specific structure is first seen on the flanks of the stage 15 embryo between the limbs [7], [8]. Instead of curling ventrally around the thorax as is the case in other vertebrates, turtle rib precursor cells grow straight into the CR resulting in the lateral extension of the shell. Several genes with described functions in mesenchyme/epithelial interactions are expressed in the CR. This observation suggests that the CR forms similarly to limb buds [6], [9]. Included in this set of genes are those encoding paracrine factors of the fibroblast growth factor (FGF), bone morphogenetic protein (BMP), and Wnt families. These are relatively small secreted proteins with demonstrated roles in developmental signaling in a wide range of organisms [6], [9]–[11].

Several lines of evidence suggest that signals from the CR are involved in the guidance of ribs into the CR of hard-shelled turtles. Local removal of the CR causes the ribs to enter adjacent regions of the CR [12], and the placement of tantalum foil between the developing ribs and the CR causes the ribs to migrate ventrally, as they do in most vertebrates [13]. The signal directing rib migration appears to be a FGF. Application of FGF inhibitors results in CR degradation and ventral rib migration suggesting an inductive role for FGFs in the CR. The application of FGF10 beads to developing chicken embryos resulted in altered rib guidance demonstrating that this process can be influenced by FGF signaling. Finally, the unusual expression of *FGF8* at the tips of *T. scripta* ribs suggests a positive feedback loop between rib expressed *FGF8* and CR expressed *FGF10*, an interaction involved in limb bud outgrowth in other species [14]. These results suggest that rib guidance in turtles relies on modifications of highly conserved FGF signaling pathways.

Similarly, ossification of the dermis between the flattened ribs forms the costal bones of the carapace and likewise appears to be mediated by well described genetic networks acting outside of their canonical vertebrate developmental compartments. The bone morphogenetic proteins (BMPs) are small secreted paracrine factors with demonstrated functions in ossification in model systems. BMPs are known to be secreted from the ribs during endochondral ossification [15]. The phosphorylation of Smad1 is a downstream event in BMP signaling. Smad1 phosphorylation in the dermis surrounding the ribs showed that BMP signaling is likely involved in turtle costal bone ossification and suggests that the ribs may be the source of these ossifying BMPs [14]. Confirmation of this hypothesis will require the development of *in situ* probes that distinguish between the various *T. scripta* BMPs.

The bones of the plastron are connected by sutures reminiscent of those that connect the facial bones of vertebrates. They appear to have their origin in a group of late migrating neural crest cells which can traced back to the neural tube at stages 16– 17 [6], [16]. The cells that produce the bones of the plastron express several molecular markers characteristic of neural crest identity including HNK-1, PDGFR-α, p75, and FoxD3 [17], [18]. Given the similar morphology of the bones and the common developmental derivation of the cells that produce these bones, homology between the plastron bones and vertebrate facial bones has been suggested [6]. The identification of the source of the cells that make up the plastron, while clarifying some questions, raises many more questions that are dependent on the development of *T. scripta* molecular markers. Gilbert et al. (2007) suggest that the skeletogenic activity of these cells may depend on the down-regulation of *Hox* genes. As is true for the *BMP* genes, the ability to determine *Hox* gene expression patterns in *T. scripta* is limited by the lack of *T. scripta* gene sequences needed to make specific RNA probes and the potential for cross-reactivity when using antibodies generated in other species.

In addition, there are several other developmental alterations in the turtle–the origin of the new musculature in the neck and around the lungs, the repositioning of the appendicular skeleton within the ribs, and the lack of a general senescence syndrome–that have not yet been investigated on a molecular level. There are limited genetic resources available for the study of turtles. Three turtle genomes (*Chrysemys* picta, *Pelodiscus sinensis*, and *Chelonia mydas*) have recently been published, although to date there is no published *T. scripta* genome [19]–[21]. A recent *T. scripta* brain transcriptome was used to support a phylogenetic grouping of turtles with the Archosaurs and significantly expanded the number of transcript sequences available for this species [22]. However, since the transcriptome was made from the brain of an adult turtle it is unlikely to contain many of the genes involved in embryonic development, many of which are expressed transiently. Genetic studies in Chelonians are difficult because turtles lay few eggs (which are available only during the breeding season) and take several years to become sexually mature. Developmental genetic studies done to date have used either antibodies from other organisms or relied on degenerate probes designed by comparing sequences from other organisms in the gene databases. In order to address the limited number of molecular markers available for working on *T. scripta* development we generated a turtle embryonic transcriptome using Illumina next generation sequencing. We used stage 14 and stage 17 embryos, an active period of induction and organogenesis, in order to ensure that genes involved in rib guidance, ossification of the carapace dermis, and early events in plastron formation would be captured in our data set. In this paper we describe the assembly and analysis of this transcriptome and identify several genes that should be useful markers for deepening our understanding of how the turtle makes its shell.

## Materials and Methods

### RNA Isolation, RNAseq Library Generation, and Next Generation Sequencing

Total RNA was isolated from stage 14 and stage 17 *T. scripta* embryos (Kleibert Alligator and Turtle Farm, Hammond LA) using TRI reagent (Sigma) according the manufacturer’s recommended protocol. RNA was quantified using a Nanodrop-2000 (Thermo Scientific) and equal amounts of RNA from each stage were combined to generate a pooled RNA sample. Two µg of the pooled total RNA sample was used to construct an Illumina sequencing library using an Illumina’s TruSeq RNA sample preparation kit (#RS-930–2001). Briefly, poly-A containing mRNA was purified from total RNA, the poly-A RNA was fragmented, double-stranded cDNA was generated from the fragmented RNA, and Illumina sequencing adapters were ligated to the ends of the fragments. The quality of the final purified library was evaluated using a BioAnalyzer 2100 automated electrophoresis system and quantified with a Qubit flourometer (Invitrogen). The library was sequenced in one 100 bp single end lane on a HiSeq 2000 (Illumina).

### Transcriptome Assembly and Analysis

The fastq file produced by the HiSeq 2000 run was assembled using the Trinity de novo transcriptome assembly package (2011-08-20 release) using default parameters except that the minimum contig length was set at 150 bp [23]. The resulting contigs were screened for vector and primer contamination using seqclean (2011-02-22 release, http://seqclean.sourceforge.net/) and the UniVec database (2011-11-21 release, http://www.ncbi.nlm.nih.gov/VecScreen/UniVec.html).

For all contigs longer than 250 bp the open reading frames most likely to encode proteins were identified using the transcripts_to_best_scoring_ORFs.pl script distributed with the 2011-10-29 release of Trinity. The 20 best BLASTP matches for each predicted protein in the NCBI nr database (downloaded 2011-10-04) were identified using a local installation of Blast2 [24]. The Blast2 output was used as the input for Blast2GO [25] to assign gene ontology and IEC enzyme codes to proteins, to map enzyme code assignments onto KEGG maps, and to identify the organismal distribution of the best Blast2 hits.

### Accession Numbers

The RNA-seq sequences have been deposited in the NCBI Sequence Read Archive as accession SRX121294 and the assembled transcripts are accessible in Genbank with accession numbers JW269948–JW501823.

### Identification of Likely Homologs

*Gallus gallus* genes were identified in the NCBI protein database and used as BLAST queries to identify putative homologs in the *T. scripta* transcriptome. Homologs from zebrafish, humans, frogs, and the anole lizard were also identified when possible. These protein sequences were aligned using the Muscle algorithm [26] implemented in MEGA5 [27]. Excessively gapped positions were removed using trimAI and were used to build maximum likelihood phylogenetic trees using MetaPIGA version 3.1 [28]. Probability consensus pruning was performed using MetaPIGA default settings with the exception of using the General Time-Reversible (GTR) model for amino acid substitutions.

### RT-PCR

RT-PCR was performed using a cDNA pool generated from RNA isolated from a stage 17 *T. scripta* embryo. Genes were amplified from the cDNA pool using Taq polymerase (NEB) for 35 cycles with a 60°C annealing temperature and a 1 minute extension time. Primers for each gene (Table 1) were designed to generate a 500–650 bp PCR product and have 65°C annealing temperatures using Primer3 [29].

**Table 1:** Primers used for RT-PCR.

FGFR1-fwd	GGCAGGCGTCTCGGAATATG
FGFR1-rev	CGGTGCCATCCACTTCACTG
Gremlin-fwd	TGCCTGGAGCATCGGTGTAA
Gremlin-rev	TGGATCTCAGGGAGCCATCC
Smad3-fwd	TGGAGGATGGCAAAGGGATG
Smad3-rev	TGTCCCTGCCTGGTCCAAAT
Sox2-fwd	TTGGCATGGAGCCCTTGAAT
Sox2-rev	CGGAAGATGGCCCAAGAGAA
FGF2-fwd	TGCCCTGGTCCAGTTTTTGG
FGF2-rev	CTGCGGGCAGCATCACCAC
BMP4-fwd	TCCGGGGAAGAGGAGGAAAG
BMP4-rev	CGTCGTGGCTGAAAGTGACC
RUNX1-fwd	TACGTGGGGGTGACCGATCT
RUNX1-rev	CCCCACACCTAACCCACGAG
HOXA7-fwd	TCTCGTTGGTCGCTGGAGTG
HOXA7-rev	ACGGGGGCTTCTCTTTTCCA
BMP5-fwd	CAGGGAGGCTTGGGAGACAA
BMP5-rev	CGATTGTGGCTTCGGTCCTT

### In Situ Hybridization

A BMP5 probe was amplified using primers tBmp5NotIR (5′- TTTGCGGCCGCTGGCTAAGGGAGGACTCT-3′) and tBmp5SalF (5′- TTTGTCGACAGGGGAGAATCACCAAAGA-3′). Whole mount stage 15 embryos were hybridized according to [30]. Briefly, embryos were fixed in 4% paraformaldehyde in PBS, rehydrated in a MeOH/PBT series, treated with proteinase K, and then washed again in PBT. Fixed embryos were probed with a digoxygenin-labeled RNA probe for BMP5 which was detected with an anti-digoxygenin alkaline phosphatase conjugated antibody.

## Results

Total RNA from stage 14 and stage 17 [7] *Trachemys scripta* embryos was prepared separately, pooled and used to generate 188,674,651 single 100 bp sequences using an Illumina HiSeq 2000. These sequences were assembled without a reference genome using the Trinity package [23] which is capable of assembling and reporting allelic variation and alternatively spliced transcripts. Trinity produced 465,923 contigs with lengths over 150 bp. In these sequences 50% of the total sequence length was contained in the 61,333 sequences longer than 757 bp. Over half of the contigs were shorter than 250 bp and most of these short sequences did not code for proteins. We decided to remove all contigs smaller than 250 bp to simplify our analysis. This left 231,876 sequences with 50% of the total sequence length contained in 37,485 sequences longer than 1166 bp. A comparison of our assembly with ten *T. scripta* developmental genes that had already been deposited in Genbank showed that the embryonic transcriptome assembly covered 98% of the existing sequences and was 99% identical to them (Table 2). Eight out of ten sequences had fewer than three differences between the existing and new sequences and four were identical. The length of the sequences in our assembly was longer than the existing sequences in every case. Assuming that the existing sequences are of high quality, these results suggest that not only is our assembly of high quality but that it also contains more complete contigs than existing Genbank sequences.

**Table 2:** Similarity between existing and new *T. scripta* sequences.

	length of existingGenbank sequence	BLASTN HSP sizes (identical/total length)	Length of embryonic transcriptome assembly sequence	% identity
EF524559.1| Trachemys scripta paired-box protein 1(Pax1) mRNA, partial cds	614	576/578	921	99.7%
EF524561.1| Trachemys scripta paired-box protein 3(Pax3) mRNA, partial cds	465	464/465	3309	99.8%
EF524562.1| Trachemys scripta twist1-like proteinmRNA, partial cds	397	393/396	2476	99.2%
EF524563.1| Trachemys scripta dermo-1 (Dermo1)mRNA, partial cds	614	447/474, 87/94	1023	94.0%
EF524564.1| Trachemys scripta engrailed 1 (En1)mRNA, partial cds	717	717/717	1548	100.0%
EF524565.1| Trachemys scripta gremlin 1 mRNA, partial cds	402	402/402	928	100.0%
EF524567.1| Trachemys scripta SRY sex determiningregion Y-box 9 (Sox9) mRNA, partial cds	340	340/340	3556	100.0%
EF527274.1| Trachemys scripta bone morphogeneticprotein 4 precursor, mRNA, partial cds	488	488/488	1775	100.0%
EF527276.1| Trachemys scripta homeobox-containingMsx2-like protein (MSX2) mRNA, partial cds	396	395/396	735	99.7%
AY327846.2|Trachemys scripta bone morphogeneticprotein 2 precursor (BMP-2) mRNA, partial cds.	1342	1273/1283	2789	99.2
Total length	5775		19060	
Average identity				99.2%

Existing *T. scripta* sequences in Genbank were used as queries in a BLASTN search of our assembled sequences. The BLAST HSP sizes represent the sizes of the sequence matches between existing sequences and new *T. scripta* transcriptome assembly sequences.

The existing *T. scripta* brain transcriptome is enriched for genes involved in nervous system function [22]. To investigate if the embryonic transcriptome is relatively enriched for genes involved in embryonic development we compared the same ten genes to the brain transcriptome sequences. Only two of these developmental genes (*En1* with a 235/717 bp match and *Sox9* with a 290/340 bp match) are represented in the brain transcriptome. Both are shorter sequences than the corresponding embryonic transcriptome sequences. The other eight sequences are not present. Comparing the two transcriptomes, 88% of all the sequences in the brain transcriptome are found in the embryonic transcriptome (with an average of 99% sequence identity and 93% coverage). Conversely, only 22% of the embryonic transcriptome sequences are found in the brain transcriptome (with an average of 99% sequence identity and 28% coverage). The larger embryonic transcriptome thus substantially increases the number of reported *T. scripta* transcript sequences and complements the existing brain transcriptome.

67,692 likely protein sequences were identified in the embryonic transcripts with an N_50_ length of 394aa. We screened these protein sequences for duplicates and identified 6,049 duplicated protein sequences resulting in 61,643 unique protein sequences. Because we sequenced RNA from multiple embryos several alleles of each gene could potentially be present in the transcriptome. Since each protein was identified from a unique assembled transcript sequence these duplicates most likely represent synonymous allelic differences or sequence variation in non-coding regions. We used Blast2GO [25] to assign gene ontology (GO) terms and Enzyme Commission (EC) numbers to each predicted protein sequence. Blast2Go analysis was based on the results of a BLASTP search of each sequence against the Genbank non-redundant (nr) protein database. Recent phylogenetic analyses have placed turtles either close to Archosaurians (crocodilians+birds) or Lepidosaurians (lizards) in the tree of life [22], [31]–[33]. One prediction about our assembly is that the protein sequences should be most similar to one of these groups of organisms. The three species with the largest absolute number of top BLASTP hits are the Chicken (*Gallus gallus*), followed by the Carolina Anole Lizard (*Anolis carolensis*) and the Zebra Finch (*Taeniopygio guttata*). Since none of these species are model systems and thus are not especially well represented in the nr database, we normalized the number of hits to the number of proteins for each species in the NCBI protein database. Using this metric, *T. scripta* protein sequences are most similar to Wild Turkey (*Meleagris gallopavo silvestris*) sequences, closely followed by the Carolina Anole Lizard. If all three bird species are combined, however, *T. scripta* proteins are most similar to the Anole lizard, followed by the birds (Table 3).

**Table 3:** Top protein hits by species.

Species	Common name	Number of top BLAST hits vs. transcriptome	Number of sequences in NCBI protein database	% of besthits	% of sequences in NCBI	ratio of best hit %/sequences in NCBI %	rank
*Gallus gallus*	Chicken	8,620	36,985	30.1%	3.4%	8.9	4
*Anolis carolinensis*	Carolina Anole lizard	5,517	17,368	19.3%	1.6%	12.2	2
*Taeniopygia guttata*	Zebra finch	4,651	17,704	16.2%	1.6%	10.1	3
*Meleagris gallopavo*	Wild turkey	4,336	13,291	15.1%	1.2%	12.5	1
*Monodelphis domestica*	Gray short-tailed opossum	2,010	20,676	7.0%	1.9%	3.7	5
*Ornithorhynchus anatinus*	Duckbill Platypus	1,087	17,735	3.8%	1.6%	2.3	6
*Xenopus laevis*	African Clawed Frog	1,398	34,431	4.9%	3.1%	1.6	7
*Homo sapiens*	Human	627	675,684	2.2%	61.7%	0.0	9
*Mus musculus*	Mouse	391	261,907	1.4%	23.9%	0.1	8
totals		28,637	1,095,781				
*Anolis carolinensis*	Carolina Anole lizard	5,517	17,368	23.9%	20.3%	1.2	1
All birds	Chicken, Zebra finch, and Wild Turkey	17,607	67,980	76.1%	79.7%	1.0	2
totals		23,124	85,348				

The number of top BLAST hits and the number of sequences in the NCBI protein database per species were used to calculate the enrichment in top hits per species relative to the number of sequences in the database. The top half of the table lists the top nine species, the bottom half compares the combined hits for all birds with the hits to the Carolina Anole Lizard.

Determining the completeness of a transcriptome in a new species is difficult because of a lack of reference genomic sequences. One prediction about a relatively complete transcriptome is that all of the major GO categories should be well represented. We assigned cellular component (CC), molecular function (MF), and biological process (BP) GO terms to each protein in the transcriptome. CC terms describe the predicted cellular location of a protein, MF terms describe the predicted function of each protein, and BP terms describe the biological pathways that proteins are predicted to participate in. All major cellular compartments, molecular functions, and biological processes are well represented in our transcriptome. Biological process annotations include 7,564 and 7,200 proteins annotated with cell communication and multicellular organism development functions, respectively (Table S1).

Another prediction about a complete transcriptome is that the enzymes that make up core metabolic pathways such as the TCA cycle should be well represented as the genes encoding these enzymes are expressed in all cells throughout development. We used Blast2Go to map each predicted protein onto the KEGG pathway database [34] which includes the TCA cycle as well as other core metabolic pathways. All of the enzymes required for the TCA cycle are represented in our transcriptome including, for example, both ADP and GDP forming Succinate CoA ligases (Table 4).

**Table 4:** TCA cycle enzymes present in the *T. scripta* developmental transcriptome.

EC number	Enzyme name	Genbank accession numbers
1.1.1.37	Malate dehydrogenase	JW457473, JW460952
1.1.1.41	Isocitrate dehydrogenase (NAD+)	JW313702, JW460801, JW464649
1.1.1.42	Isocitrate dehydrogenase (NADP+)	JW315818, JW459818, JW460815
1.2.4.1	Pyruvate dehydrogenase (acetyl-transferring)	JW458559, JW463459
1.2.4.2	Oxoglutarate dehydrogenase (succinyl-transferring)	JW425409, JW443178, JW460829, JW489499, JW460830, JW460831
1.3.5.1	Succinate dehydrogenase (ubiquinone)	JW460432, JW463499
1.3.99.1	Succinate dehydrogenase	JW317082, JW461916
1.8.1.4	Dihydrolipoyl dehydrogenase	JW459096
2.3.1.12	Dihydrolipoyllysine-residue acetyltransferase	JW313827
2.3.1.61	Dihydrolipoyllysine-residue succinyltransferase	JW464483
2.3.3.1	Citrate (Si)-synthase	JW458401, JW459037
2.3.3.8	ATP citrate synthase	JW305869, JW460741, JW460742
4.1.1.32	Phosphoenolpyruvate carboxykinase (GTP)	JW288259, JW288260, JW461270, JW461271
4.1.3.6	Citrate (pro-3S)-lyase	JW402608, JW460741, JW460742
4.2.1.2	Fumarate hydratase	JW319039
4.2.1.3	Aconitate hydratase	JW321248, JW461661
6.2.1.4	Succinate–CoA ligase (GDP-forming)	JW305869, JW310451
6.2.1.5	Succinate–CoA ligase (ADP-forming)	JW305869, JW460741, JW463477, JW460742
6.4.1.1	Pyruvate carboxylase	JW314460

Predicted proteins in the transcriptome were mapped to the TCA KEGG metabolic pathway using Blast2Go.

**Figure 1:**
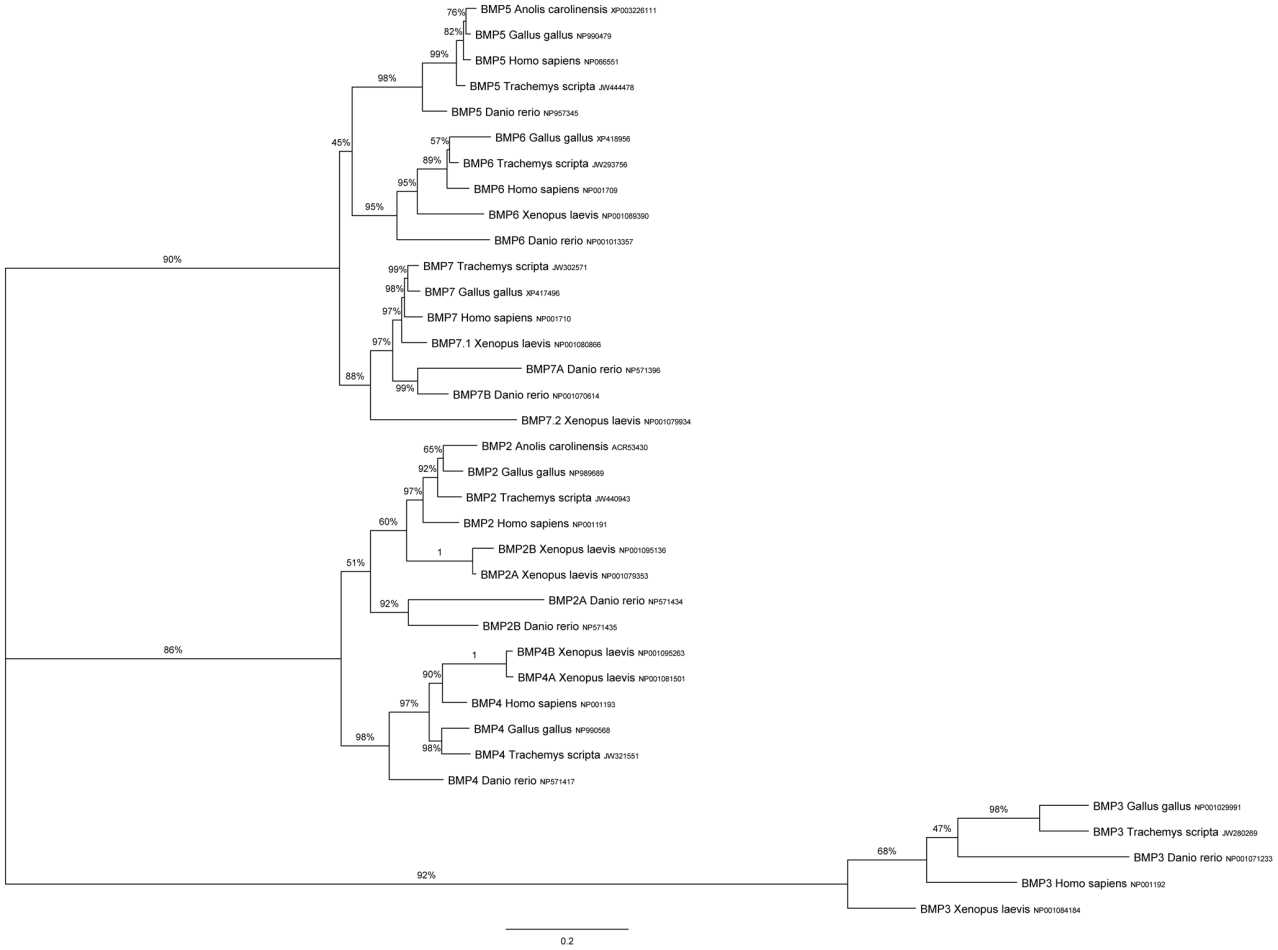
Identification of *T. scripta BMP2*-*7* genes. The *T. scripta* transcriptome was queried with BMP protein sequences from other organisms. Sequences were aligned and excessively gapped positions were removed (final size of dataset = 285aa/species). Their ML relationships were inferred using MetaPIGA. Labels on nodes indicate posterior probabilities. Scale bar units are the number of amino acid substitutions per site. Accession numbers are to the right of each sequence name.

In order for the sequences in our transcriptome to serve as a useful resource for turtle developmental biologists they must enable the identification of homologues in other organisms and the generation of *in situ* probes. To demonstrate that our transcriptome can be used to identify homologs of developmentally important genes we queried the transcriptome with developmental protein sequences from several species (chicken, zebrafish, humans, frogs, and the anole lizard when possible). Several of the genes we were interested in identifying (e.g., *BMP*s and *FGF*s) are members of gene families. For genes in these families, we identified multiple transcripts for each query. To determine the placement of each transcript within the gene family we constructed phylogenetic trees based on protein sequence similarity of all of the gene family members we identified. In most cases, it was possible to determine which family member each turtle transcript was most similar to, and in most cases the *T. scripta* transcriptome contains complete or nearly complete coverage of all members of each gene family. As an example, one of the gene families we investigated was the *BMP* family which has been implicated in ossification of the carapace. We used BMP2-7 sequences from a range of vertebrates to query the transcriptome. In each case we identified a single *T. scripta* gene which clusters with family members from other species (Fig. 1).

**Figure 2:**
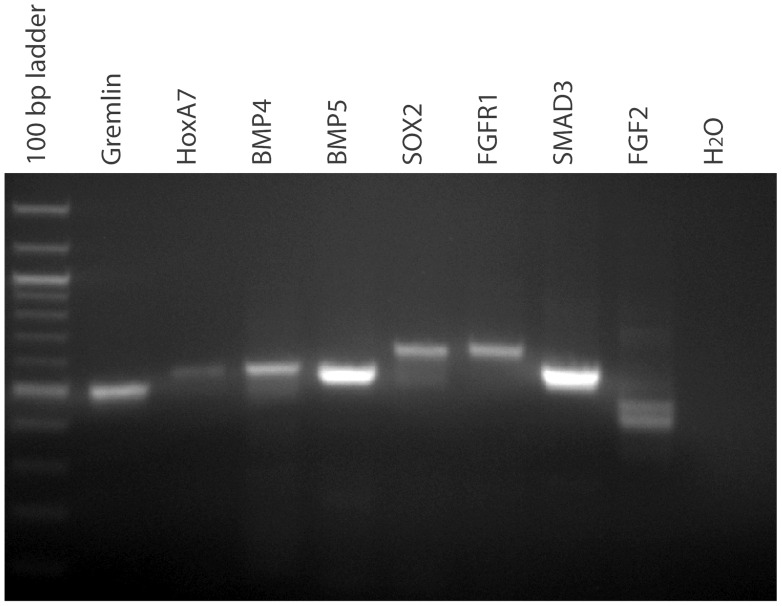
RT-PCR of developmentally important genes from a stage 17 *T. scripta* cDNA pool.

To investigate if the transcriptome sequences could be used to amplify probes for use in *in situ* experiments we selected nine developmental genes, *Gremlin*, *HoxA7*, *BMP4*, *BMP5*, *SOX2*, *RUNX1*, *FGFR1*, *SMAD3*, and *FGF2* (accession numbers JW357402,JW364078, JW321551, JW444478, JW460170, JW373558, JW459374, JW388739, and JW429145) and designed PCR primers to amplify each from a stage 17 cDNA pool. Using standard PCR conditions all of the genes apart from *RUNX1* amplified and each produced a single dominant product except for *FGF2* which produced two bands (Figure 2). It is possible that the *RUNX1* primers did not amplify a fragment because it is not expressed at stage 17. The amplification of a single dominant product in seven out of nine targets on the first try (a 77% success rate) is much more efficient than degenerate PCR approaches for probe production which often require extensive optimization.

**Figure 3:**
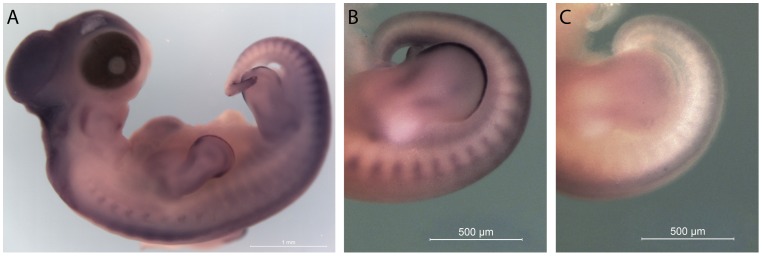
*BMP5* expression in a stage 15 *T. scripta* embryo. *BMP5* expression is associated with the developing vertebrae in the cervical region and the newly formed somites in the tailbud. In addition, BMP5 is expressed in the anterior and posterior margins of the autopod, and in the apical ectodermal ridge of the developing limb buds (A and B antisense, C sense).

Finally, a *BMP5* probe was designed based on the predicted *T. scripta* sequence and used as an in situ probe on a stage 15 embryo. *BMP5* expression is associated with the developing vertebrae in chicks and mice, and it is important in determining the curvature of the rib [35]–[38]. In addition to this conserved expression pattern in the vertebrae, turtle *BMP5* is also expressed in the apical ectodermal ridges of the embryonic limb buds and in the margin mesoderm surrounding them (Fig. 3). This limb bud expression has not been reported in chicks or mice [39]–[41], suggesting an additional developmental role for this conserved gene in turtles.

## Discussion

Understanding *T. scripta* development including the development of the plastron and carapace has been limited by a lack of genomic resources. Few sequences important for the study of embryonically expressed developmental genes were available before this study. We have used a next generation sequencing approach to assemble a high quality *T. scripta* transcriptome without a reference genome. These sequences were assigned putative functional annotations based on the predicted translation products. GO categories include all core cellular and molecular processes suggesting that the transcriptome is relatively complete for these functions. Classes of genes which are not expressed during the developmental stages we sampled would not be represented in this transcriptome.

We demonstrated that the sequences generated in this study can be used to design PCR primers with which we can amplify important developmental genes. This resource enables the design of *in situ* probes without resorting to degenerate PCR. We have used these sequences to design a *BMP5* probe. The probe detects *BMP5* expression both in expected locations in *T. scripta* embryos (vertebrae), but also in an unexpected location (the anterior limb buds). Further study of these expression patterns may shed light not only on shell development but also on other unique and previously undescribed mechanisms of turtle development.

The placement of turtles in the tree of life is controversial. Different data sets and methodologies, even from the same authors, result in different placements. Turtles have been grouped both with the lizards (Lepidosaurs) and with birds and crocodiles (Archosaurs), generally depending on whether morphological or molecular characters, respectively, were analyzed [22], [31]–[33]. A simple analysis of our transcriptome sequences shows that they are very similar to both lizard and bird sequences, consistent with either grouping. Given the limitations of both our transcriptome (it samples a limited set of developmental stages) and bird and lizard sequences, neither of which are ‘complete’, it seems unlikely that a more sophisticated analysis performed using our data will resolve this ongoing controversy.

We hope that this transcriptome provides a valuable resource for the *T. scripta* community both for developmental studies as well as for genome annotation in the future and is of use to other biologists interested in comparative genomics.

## Supporting Information

Table S1 **Cellular component (CC), molecular function (MF), and biological process (BP) GO categories assigned to proteins identified in the** ***T. scripta*** **embryonic transcriptome.**
(XLSX)

## References

[1] Rowan BA, Weigel D, Koenig D (2011) Developmental genetics and new sequencing technologies: the rise of nonmodel organisms. Dev Cell 21: 65–76. 21763609 10.1016/j.devcel.2011.05.021

[2] Gilbert SF (2009) The Adequacy of Model Systems for Evo-Devo: Modeling the Formation of Organisms. In: Barberousse A, Morange M, Pradeu T, editors. Modeling the Formation of Society Mapping the Future of Biology: Springer Netherlands. 57–68.

[3] Milinkovitch MC, Tzika A (2007) Escaping the mouse trap: the selection of new Evo-Devo model species. J Exp Zool B Mol Dev Evol 308: 337–346. 17520701 10.1002/jez.b.21180

[4] Gaffney ES, Hutchison JH, Jenkins FA Jr, Meeker LJ (1987) Modern turtle origins: the oldest known cryptodire. Science 237: 289–291. 17772056 10.1126/science.237.4812.289

[5] Gaffney ES (1990) The comparative osteology of the Triassic turtle Proganochelys. Bulletin of the AMNH. 1–263.

[6] Gilbert SF, Cebra-Thomas JA, Burke AC (2007) How the Turtle Gets Its Shell. In: Wyneken J, Godfrey MH, Bels V, editors. Biology of Turtles: From Structures to Strategies of Life. Boca Raton, FL: CRC Press. 1–16.

[7] Greenbaum E (2002) A standardized series of embryonic stages for the emydid turtle Trachemys scripta. Can J Zool 80: 1350–1370.

[8] Yntema CL (1968) A series of stages in the embryonic development of Chelydra serpentina. J Morphol 125: 219–251. 5681661 10.1002/jmor.1051250207

[9] Kawashima-Ohya Y, Narita Y, Nagashima H, Usuda R, Kuratani S (2011) Hepatocyte growth factor is crucial for development of the carapace in turtles. Evol Dev 13: 260–268. 21535464 10.1111/j.1525-142X.2011.00474.x PMC3121961

[10] Kuraku S, Usuda R, Kuratani S (2005) Comprehensive survey of carapacial ridge-specific genes in turtle implies co-option of some regulatory genes in carapace evolution. Evol Dev 7: 3–17. 15642085 10.1111/j.1525-142X.2005.05002.x

[11] Moustakas JE (2008) Development of the carapacial ridge: implications for the evolution of genetic networks in turtle shell development. Evol Dev 10: 29–36. 18184355 10.1111/j.1525-142X.2007.00210.x

[12] Yntema CL (1970) Extirpation experiments on embryonic rudiments of the carapace of Chelydra serpentina. J Morphol 132: 235–243. 5482414 10.1002/jmor.1051320209

[13] Burke AC (1989) Development of the turtle carapace: Implications for the evolution of a novel bauplan. J Morphol 199: 363–378. 10.1002/jmor.1051990310 29865619

[14] Cebra-Thomas J, Tan F, Sistla S, Estes E, Bender G, et al (2005) How the turtle forms its shell: a paracrine hypothesis of carapace formation. J Exp Zool B Mol Dev Evol 304: 558–569. 15968684 10.1002/jez.b.21059

[15] Minina E, Kreschel C, Naski MC, Ornitz DM, Vortkamp A (2002) Interaction of FGF, Ihh/Pthlh, and BMP signaling integrates chondrocyte proliferation and hypertrophic differentiation. Dev Cell 3: 439–449. 12361605 10.1016/s1534-5807(02)00261-7

[16] Greenbaum E, Carr JL (2002) Staging criteria for embryos of the spiny softshell turtle, Apalone spinifera (Testudines: Trionychidae). J Morphol 254: 272–291. 12386898 10.1002/jmor.10036

[17] Clark K, Bender G, Murray BP, Panfilio K, Cook S, et al (2001) Evidence for the neural crest origin of turtle plastron bones. Genesis 31: 111–117. 11747201 10.1002/gene.10012

[18] Cebra-Thomas JA, Betters E, Yin M, Plafkin C, McDow K, et al (2007) Evidence that a late-emerging population of trunk neural crest cells forms the plastron bones in the turtle Trachemys scripta. Evol Dev 9: 267–277. 17501750 10.1111/j.1525-142X.2007.00159.x

[19] K G (2009) Genome 10K: A Proposal to Obtain Whole-Genome Sequence for 10,000 Vertebrate Species. Journal of Heredity 100: 659–674. 19892720 10.1093/jhered/esp086 PMC2877544

[20] Wang Z, Pascual-Anaya J, Zadissa A, Li W, Niimura Y, et al.. (2013) The draft genomes of soft-shell turtle and green sea turtle yield insights into the development and evolution of the turtle-specific body plan. Nat Genet. 10.1038/ng.2615 PMC4000948 23624526

[21] Abramyan J, Badenhorst D, Biggar KK, Borchert GM, Botka CW, et al (2013) The western painted turtle genome, a model for the evolution of extreme physiological adaptations in a slowly evolving lineage. Genome Biol 14: R28. 23537068 10.1186/gb-2013-14-3-r28 PMC4054807

[22] Tzika AC, Helaers R, Schramm G, Milinkovitch MC (2011) Reptilian-transcriptome v1.0, a glimpse in the brain transcriptome of five divergent Sauropsida lineages and the phylogenetic position of turtles. Evodevo 2: 19. 21943375 10.1186/2041-9139-2-19 PMC3192992

[23] Grabherr MG, Haas BJ, Yassour M, Levin JZ, Thompson DA, et al (2011) Full-length transcriptome assembly from RNA-Seq data without a reference genome. Nat Biotechnol 29: 644–652. 21572440 10.1038/nbt.1883 PMC3571712

[24] Altschul SF, Gish W, Miller W, Myers EW, Lipman DJ (1990) Basic local alignment search tool. J Mol Biol 215: 403–410. 2231712 10.1016/S0022-2836(05)80360-2

[25] Conesa A, Gotz S, Garcia-Gomez JM, Terol J, Talon M, et al (2005) Blast2GO: a universal tool for annotation, visualization and analysis in functional genomics research. Bioinformatics 21: 3674–3676. 16081474 10.1093/bioinformatics/bti610

[26] Edgar RC (2004) MUSCLE: multiple sequence alignment with high accuracy and high throughput. Nucleic Acids Res 32: 1792–1797. 15034147 10.1093/nar/gkh340 PMC390337

[27] Tamura K, Peterson D, Peterson N, Stecher G, Nei M, et al (2011) MEGA5: molecular evolutionary genetics analysis using maximum likelihood, evolutionary distance, and maximum parsimony methods. Mol Biol Evol 28: 2731–2739. 21546353 10.1093/molbev/msr121 PMC3203626

[28] Helaers R, Milinkovitch MC (2010) MetaPIGA v2.0: maximum likelihood large phylogeny estimation using the metapopulation genetic algorithm and other stochastic heuristics. BMC Bioinformatics 11: 379. 20633263 10.1186/1471-2105-11-379 PMC2912891

[29] Rozen S, Skaletsky H (2000) Primer3 on the WWW for general users and for biologist programmers. Methods Mol Biol 132: 365–386. 10547847 10.1385/1-59259-192-2:365

[30] Wilkinson DG (1993) Whole-mount in situ hybridization of vertebrate embryos. In: Wilkinson DG, editor. In situ Hybridization: A Practical Approach Oxford: IRL Press. 75–83.

[31] Lyson TR, Bever GS, Bhullar BA, Joyce WG, Gauthier JA (2010) Transitional fossils and the origin of turtles. Biol Lett 6: 830–833. 20534602 10.1098/rsbl.2010.0371 PMC3001370

[32] Lyson TR, Sperling EA, Heimberg AM, Gauthier JA, King BL, et al (2012) MicroRNAs support a turtle+lizard clade. Biol Lett 8: 104–107. 21775315 10.1098/rsbl.2011.0477 PMC3259949

[33] Crawford NG, Faircloth BC, McCormack JE, Brumfield RT, Winker K, et al.. (2012) More than 1000 ultraconserved elements provide evidence that turtles are the sister group of archosaurs. Biol Lett. 10.1098/rsbl.2012.0331 PMC3440978 22593086

[34] Kanehisa M, Goto S (2000) KEGG: Kyoto Encyclopedia of Genes and Genomes. Nucleic Acids Research 28: 27–30. 10592173 10.1093/nar/28.1.27 PMC102409

[35] Darnell DK, Kaur S, Stanislaw S, Davey S, Konieczka JH, et al (2007) GEISHA: an in situ hybridization gene expression resource for the chicken embryo. Cytogenet Genome Res 117: 30–35. 17675842 10.1159/000103162

[36] Green EL, Green MC (1946) Effect of the short ear gene on number of ribs and presacral vertebrae in the house mouse. Am Nat 80: 619–625. 20284341 10.1086/281482

[37] Guenther C, Pantalena-Filho L, Kingsley DM (2008) Shaping skeletal growth by modular regulatory elements in the Bmp5 gene. PLoS Genet 4: e1000308. 19096511 10.1371/journal.pgen.1000308 PMC2592695

[38] Storm EE, Kingsley DM (1996) Joint patterning defects caused by single and double mutations in members of the bone morphogenetic protein (BMP) family. Development 122: 3969–3979. 9012517 10.1242/dev.122.12.3969

[39] Solloway MJ, Robertson EJ (1999) Early embryonic lethality in Bmp5;Bmp7 double mutant mice suggests functional redundancy within the 60A subgroup. Development 126: 1753–1768. 10079236 10.1242/dev.126.8.1753

[40] Zuzarte-Luis V, Montero JA, Rodriguez-Leon J, Merino R, Rodriguez-Rey JC, et al (2004) A new role for BMP5 during limb development acting through the synergic activation of Smad and MAPK pathways. Dev Biol 272: 39–52. 15242789 10.1016/j.ydbio.2004.04.015

[41] Geetha-Loganathan P, Nimmagadda S, Huang R, Scaal M, Christ B (2006) Expression pattern of BMPs during chick limb development. Anat Embryol (Berl) 211 Suppl 187–93. 10.1007/s00429-006-0129-6 17024298

